# Human Gut Microbiota in Coronary Artery Disease: A Systematic Review and Meta-Analysis †

**DOI:** 10.3390/metabo12121165

**Published:** 2022-11-23

**Authors:** Marcin Choroszy, Kamil Litwinowicz, Robert Bednarz, Tomasz Roleder, Amir Lerman, Takumi Toya, Karol Kamiński, Emilia Sawicka-Śmiarowska, Magdalena Niemira, Beata Sobieszczańska

**Affiliations:** 1 Department of Microbiology, Wroclaw Medical University, 50-367 Wroclaw, Poland; 2 Department of Biochemistry and Immunochemistry, Wroclaw Medical University, 50-367 Wroclaw, Poland; 3 Ninewells Hospital and Medical School, James Arrott Drive, Dundee DD1 9SY, UK; 4 Research and Development Centre, Regional Specialist Hospital, 51-124 Wroclaw, Poland; 5 Department of Cardiovascular Medicine, Mayo Clinic, Rochester, MN 55905, USA; 6 Division of Cardiology, National Defense Medical College, Tokorozawa 359-8513, Japan; 7 Department of Population Medicine and Lifestyle Diseases Prevention, Medical University of Bialystok, 15-269 Bialystok, Poland; 8 Department of Cardiology, Medical University of Bialystok, 15-089 Bialystok, Poland; 9 Clinical Research Centre, Medical University of Bialystok, 15-089 Bialystok, Poland

**Keywords:** coronary artery disease, atherosclerosis, gut microbiome, dysbiosis, SCFA, LPS

## Abstract

In recent years, the importance of the gut microbiome in human health and disease has increased. Growing evidence suggests that gut dysbiosis might be a crucial risk factor for coronary artery disease (CAD). Therefore, we conducted a systematic review and meta-analysis to determine whether or not CAD is associated with specific changes in the gut microbiome. The V3–V4 regions of the 16S rDNA from fecal samples were analyzed to compare the gut microbiome composition between CAD patients and controls. Our search yielded 1181 articles, of which 21 met inclusion criteria for systematic review and 7 for meta-analysis. The alpha-diversity, including observed OTUs, Shannon and Simpson indices, was significantly decreased in CAD, indicating the reduced richness of the gut microbiome. The most consistent results in a systematic review and meta-analysis pointed out the reduced abundance of *Bacteroidetes* and *Lachnospiraceae* in CAD patients. Moreover, *Enterobacteriaceae*, *Lactobacillus*, and *Streptococcus* taxa demonstrated an increased trend in CAD patients. The alterations in the gut microbiota composition are associated with qualitative and quantitative changes in bacterial metabolites, many of which have pro-atherogenic effects on endothelial cells, increasing the risk of developing and progressing CAD.

## 1. Introduction

Various studies on coronary atherosclerosis have revealed that chronic vascular inflammation, which begins with a coronary endothelial injury, is an essential process in the development of coronary artery disease (CAD) [1]. Despite advances in various drug therapies and interventions, CAD remains one of the most common causes of death worldwide [2]. In recent years, data from rigorous preclinical studies and epidemiological studies linking changes in the gut microbiome to vascular endothelial dysfunction and vascular inflammation have accumulated [3].

The gut microbiome comprises over 2000 species, most of which fall into the four main phyla: *Bacteroidetes, Firmicutes, Proteobacteria*, and *Actinobacteria* [4]. A diverse, well-balanced gut microbiota is crucial to human health. Gut microbiota consisting of a 100,000,000,000,000 microbes living in a symbiotic host relationship regulate host metabolism, insulin sensitivity, intestinal endocrine, and neurological function. Gut bacteria produce neurotransmitters, influence the maturation and training of the host immune system, neutralize exogenous toxins, and protect against overgrowth pathogens [5,6,7]. Active microbial metabolites, such as short-chain fatty acids, vita-mins, autoinducers, indole derivates, bile acid metabolites, microbial amino acids, and polyamines, directly regulate human physiology [8].

Disruption of the diversity and abundance of the gut microbiota, termed gut dysbiosis, underlies many diseases, including metabolic syndrome associated with obesity and diabetes. Gut dysbiosis underlies inflammatory bowel disease (IBD), irritable bowel disease (IBS), colon cancer, asthma, autism, Parkinson’s disease, Alzheimer’s disease, schizophrenia, depression and cardiovascular diseases [5,9,10,11,12]. An imbalance in the abundance of specific bacterial taxa in the gut microbiota correlates with a deficiency or excess of bacterial metabolites that fundamentally affect the physiological status of the host cells, including endothelial cells. To exemplify, specific tryptophan-derived gut microbiota metabolites, such as indole metabolites and butyrate, serve as regulators of the intestinal barrier, increasing the expression of tight junction proteins that ensure the tightness of the epithelial barrier [13,14,15]. Hence, the depletion of butyrate-producing bacterial taxa, e.g., *Lachnospiraceae*, may compromise intestinal barrier integrity and leakage of bacterial metabolites into the bloodstream [16]. One of the most critical consequences of intestinal barrier dysfunction in gut dysbiosis is endotoxemia and the activation of immune processes leading to chronic subacute inflammation [17,18]. Apart from immune cells, low-grade endotoxemia affects endothelial cells, contributing to the initiation and progression of atherosclerosis [19,20]. Many studies have shown alterations in the composition and diversity of the gut microbiota in CAD [21].

Nevertheless, it is still uncertain which bacterial taxa are altered in CAD and whether these alterations are consistent across different study populations. Studies of gut microbiota composition are always affected by factors related to the study population, such as age, diet, chronic diseases, medications, and physical activity [22]. In addition, gut microbiota studies are complicated by the choice of sequencing methods and bioinformatic pipelines, which significantly influence the results [23].

A recent study on simulated microbiome datasets highlights the importance of using consistent analytical pipelines. Nearing et al. [24] have shown that the appropriate tools for the differential abundance of microbial taxa testing impact the result. Therefore, this meta-analysis and systematic review aimed to provide an overview of the literature linking changes in gut microbiota with CAD using consistent analytical tools across all the datasets.

## 2. Materials and Methods

### 2.1. Systematic Review

This systematic review is registered in the International Prospective Register of Systematic Reviews “PROSPERO” ID CRD42020187549. The study was conducted following the Preferred Reporting Items of Systematic reviews and Meta-Analyses (PRISMA) guidelines.

#### 2.1.1. Search Strategy

A comprehensive literature search was performed. The search terms were “(Human Microbiomes OR Human Microbiome OR Human Microflora OR Microbial Community Structure OR Human Flora OR Composition, Microbial Community) AND (atherosclerosis or atherogenesis or Coronary atherosclerosis or Coronary Artery Disease or Coronary Atherosclerosis) AND ischemic heart disease NOT Review” for Medline on PubMed. The search terms were appropriately modified for other databases and are included in the Supplementary Materials (Supplementary S1). The search strategy was applied to the following databases: PubMed, Scopus, Web of Science, Embase, CINAHL, and Cochrane. We used Google Scholar to search for grey literature; the last search was conducted on 14 December 2021. Before proceeding with the selection of eligible studies, all duplicates were removed.

#### 2.1.2. Inclusion and Exclusion Criteria

We have included studies that analyzed the differences in the profile of the intestinal microbiome between patients with CAD (both stable and unstable) and healthy controls. Only human studies were included, and neither publication status nor language restrictions were imposed. The primary outcome measure was a comparison of relative abundances of bacterial phyla, families, and genera of human gut microflora. We excluded papers in which CAD was diagnosed by clinical symptoms or ECG only, without coronary imaging methods. Articles in which the control group included subjects with coronary artery disease, or the study group consisted of cadavers, were also excluded.

#### 2.1.3. Assessment of Eligibility and Data Extraction

**Figure 1:**
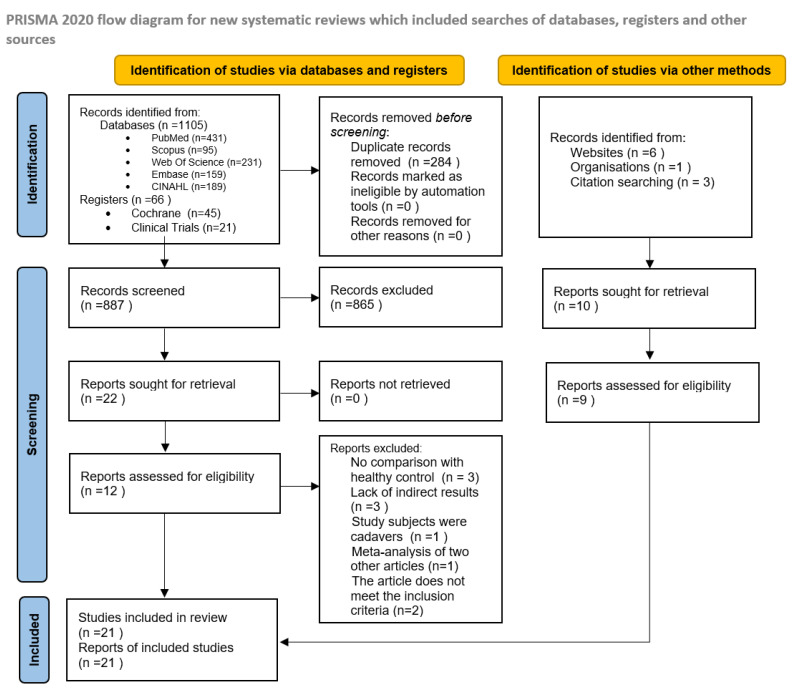
Flowchart of literature search according to Preferred Reporting Items for Systematic Reviews and Meta-Analyses guidelines.

After removing the duplicates, two authors (MC and RB) independently screened obtained studies by titles and abstracts for relevance to the topic of our systematic review. The studies obtained by screening were read in full text, and eligibility was determined based on inclusion and exclusion criteria. The records that did not fulfill the criteria were removed without using automatic tools. Discrepancies were discussed, and a third author (KL) arbitrated if disagreement was not resolved. We have included peer-reviewed papers and those still awaiting review (grey literature). The eligibility assessment of studies is summarized in Figure 1. The study quality was assessed using a modified Newcastle Ottawa Scale (Supplementary).

### 2.2. Meta-Analysis

The meta-analytical pipeline has been run using Snakemake version 6.6.1. [25]. The characteristics of the studies included in the meta-analysis and systematic review are provided in Table 1. Only studies that provided raw sequences from 16S rRNA sequencing were included in the meta-analysis. The results were obtained by analyzing the samples using two approaches. First, all datasets were analyzed separately, and their results were combined in the random-effects meta-analysis (single study approach). In the second approach, all the samples were analyzed as one combined dataset (combined studies approach).

#### 2.2.1. Readings Preparation and zOTU Picking

We have removed primers using cutadapt [26]. Primers used for the single study approach are provided in the table available in the supplementary. For the combined study approach, we have used 338F (5′-CTACGGGNGGCWGCAG-3′) and 805R (5′-GACTACHVGGGTATCTAATCC-3′) primers, except the Mayo dataset where 926R (5′-CCGTCAATTCMTTTRAGT-3′) was used. Sawicka-Smiarowska dataset [27] was excluded from the combined study approach due to the choice of primers in the study, which did not allow for obtaining sequences that were globally aligned with other studies. Next, all of the readings were truncated to 250 bps leaving us with globally trimmed data (e.g., all reads starting and ending at the same position). Furthermore, reading preparation and zOTU picking have been performed using usearch v11.0.667 and UNOISE. zOTU refers to correct biological sequences with a 100% identity threshold [28,29]. The taxonomy was resolved using DECIPHER IDTAXA [30] with Genome Taxonomy Database (version 06-RS202) [31] as a reference. For downstream analyses, the data have been imported into the phyloseq object [32] and filtered to leave only zOTUs present in at least 20% of the samples and with at least five counts.

#### 2.2.2. Differential Abundance Testing

Differential abundance (DA) testing was performed using three approaches: (1) DESeq2, which models the data using the negative binomial distribution, (2) MetagenomeSeq, which uses a zero-inflated Gaussian model, and (3) ANCOM-BC, which uses an offset-based log-linear model and currently appears to be the best method for controlling the false-discovery ratio without losing statistical power. Only bacteria detected as statistically significant metagenomeSeq, Ancom-BC, or DESeq2 in at least three of 7 studies were included. A comprehensive discussion of the DA testing approaches is out of the scope of our manuscript, and we refer the reader to the large body of research already published on the topic [33]. For all the approaches, we have assumed a significance level of *p* < 0.05. We have used the Benjamini and Hochberg method to adjust for multiple testing.

#### 2.2.3. Statistical Analysis

Alpha diversity indices (observed zOTUs, Fischer, Chao1, Shannon, ACE, and Simpson indices) were calculated on data rarefied to the depth of 5000. Beta diversity was assessed using Unweighted UniFrac. The statistical significance was determined using the Wilcoxon test for the alpha diversity and PERMANOVA with 999 permutations for the beta diversity. PERMANOVA was run with the vegan R package [34]. The single study approach calculated relative abundances using the mean difference in centered log ratios (CLR). The obtained relative abundances were used to estimate odds ratios using Agresti’s generalized odd ratios [35] as implemented in the genodds package [36]. Next, odd ratios were summarized in a random-effects meta-analysis performed using the meta R package [37].

## 3. Results

**Figure 2:**
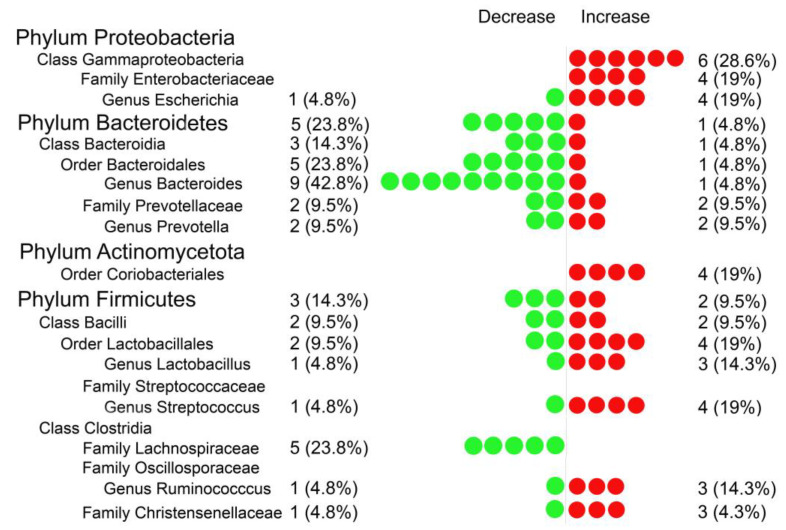
The results of a systematic review. The red circles describe the percentage of articles with increased bacterial relative abundance in CAD patients. Oppositely, green circles describe the percentage of articles with decreased bacterial relative abundance in CAD patients. One circle represents one study analyzed.

Our search has yielded 1181 articles. After removing the duplicates, 897 unique studies were identified, out of which 21 met inclusion criteria for systematic review. Among these 21 articles, 7 had raw data available and could be used to conduct a meta-analysis. (Figure 1). In the systematic review, the relative abundance of gut microbiota from all 21 eligible papers were compared. Bacterial taxa detected as significant in at least four studies are included in Figure 2. In the meta-analysis, only bacteria detected as significant in at least three of the seven eligible articles were reported. The complete results of the systematic review are provided in Table 1, and the characteristics of studies included in the meta-analysis are available in the Supplement.

### 3.1. Characteristics of Included Studies

Many factors may affect the gut microbiome composition. We compared all eligible studies by the following: age, gender, BMI, and diabetes mellitus. The complete results are provided in the Supplement.

The average age of participants was between the sixth and seventh decade of life. In five articles, differences in age between groups were significant; in 13 other articles were insignificant, and there were no available data for comparison in three articles.

In the majority of studies, participants were male. In five articles, differences in gender between groups were significant, ten others were insignificant, and six articles had no available data for comparison. The BMI of participants generally had values typical for overweight (BMI = 25–30). In four articles, differences in BMI were significant, in ten the differences were insignificant, and in seven, there were no available data for comparison. There were relatively limited data on the prevalence of diabetes in participants. In four articles, differences in diabetes were significant, in five the differences were insignificant, and in 12, there were no available data for comparison.

### 3.2. Systematic Review Results

A comprehensive comparison between CAD patients and controls regarding the relative abundance at the phylum level demonstrated changes related to four phyla, i.e., Proteobacteria, *Bacteroidetes*, Actinomycetota, and Firmicutes. The increase of Gammaproteobacteria has been reported in six (28.6%) studies and the rise of the *Enterobacteriaceae* family within the Proteobacteria phylum in four (19%) studies in CAD patients. Moreover, four (19%) studies recorded the rise of the Escherichia genus of the *Enterobacteriaceae* family. Overall, seven (33.3%) articles reported an increased abundance of Gammaproteobacteria taxa (Figure 2, Table 1).

*Bacteroidetes* phylum decline in CAD was reported in five (23.8%) studies (Table 1; Figure 2). On the contrary, one cross-sectional study reported an increased abundance of *Bacteroidetes* in CAD compared to the control group. Remarkably, at the genus level, a decrease in the relative abundance of Bacteroides in CAD has been recorded in nine (42.8%) studies. Overall, a decline in the relative abundance of the *Bacteroidetes* taxa was recorded in 16 (76.2%) of all 21 articles analyzed, making the decrease in these taxa strikingly CAD-related. Furthermore, conflicting results regarding the Prevotellaceae family and Prevotella genus within the Bacteroidales order were associated with CAD, where the relative abundance of these taxa were decreased in two (9.5%) studies and increased in two other studies.

The results regarding the Firmicutes phylum were conflicting. In two (9.5%) studies, the relative abundance of Firmicutes was increased, whereas in three other (14.3%) decreased in CAD compared to the control group. Within the Firmicutes phylum, there were conflicting results concerning the Bacilli class. In two (9.5%) studies, the taxon was increased, whereas in two others decreased in CAD compared to the control group. However, within the Bacilli taxon, the increased relative abundance of the *Lactobacillales* order was associated with CAD patients in four (19%) studies and the *Lactobacillus* genus in three (14.3%) studies in contrast to the control group. Moreover, within the Firmicutes phylum, the decrease in the relative abundance of the *Lachnospiraceae* family of class Clostridia in CAD patients versus the control group was demonstrated in five (23.8%) studies. On the contrary, within the Firmicutes phylum, the Christensenellaceae family abundance was increased in CAD in three (14.3%) studies. The systematic review analysis also demonstrated the increased relative abundance within the Streptococcaceae family and the *Streptococcus* genus among CAD patients in four (19%) studies compared to the control group. These results indicate a quantitative disturbance within the Firmicutes, mainly concerning an increase in the abundance of *Lactobacillus* and *Streptococcus*, as well as bacteria from the Christensenellaceae family, with a concomitant decrease in the abundance of bacteria from the *Lachnospiraceae* family.

The less prominent changes in the gut microbiota of CAD patients concerned the Coriobacteriales order of the phylum Actinobacteria, which was decreased in four (19%) studies in CAD patients. Changes in the abundance of other bacterial groups, i.e., Desulfovibrio, Parabacteroides, and Fusobacterium in CAD, were recorded in only a few articles (Table 1).

**Table 1:** Key characteristics of included studies. Legend: RoB—the risk of bias, NOS—Newcastle-Ottawa Scale, “+” significant difference in diversity, “−” no significant difference in diversity, PCI—percutaneous coronary intervention, CABG—coronary artery bypass graft, IBD—inflammatory bowel disease, GIT—gastrointestinal tract, ACS—acute coronary syndrome, UA—unstable angina, SA—stable angina, CAD—coronary artery disease, Ctrl—control, “▲”increased relative abundance in CAD patients, “▼“ decreased relative abundance in CAD patients.

**RoB** **NOS**	7	10	7	10	8
**Diversity** **α β**	−	No data	+	+	No data
	−	No data	−	+	No data
**Major outcome-** **CAD relative abundance**	*Bacteroidales*▼ *Gammaproteobacteria*▲ *Enterobacteriaceae*▲ *Prevotella* ▲	*Lactobacillales* ▲ *Bacteroides* ▼	*Gammaproteobacteria*▲ *Firmicutes*▼ *Lactobacillales*▼ *Enterobacteriaceae* ▲ *Lachnospiraceae*▼ *Prevotella*▼ *Bacilli*▼ *Christensellaceae*▲ *Prevotellaceae ▼*	*Bacteroidetes*▲ *Firmicutes* ▼ *Bacteroidales*▲ *Coriobacteriales*▲ *Christensellaceae*▲ *Prevotellaceae*▲	*Enterobacteriaceae* ▲ *Escherichia/Shigella* ▲
**Major exclusion criteria**	patients less than 19 years, pregnancy abnormal kidney function	Patients with systemic diseases: hepatic, renal, collagen, malignancy	other identifiable etiologies of coronary thrombi, active infection during admission.	heart failure, structural heart disease, history of antibiotic use within 1 month, serious dysfunction of liver or kidney	antibiotics, probiotics, decompensated chronic diseases, oncological diseases
**Major inclusion criteria** **CAD Control**	No prior heart attacks or strokes, and no antibiotic use within three months before enrollment	-coronary risk factors: hypertension, diabetes, and/or dyslipidemia,	no previous history of cardiovascular disease, no evidence of active infection	coronary arteries without stenosis	over 50 years, without cardiovascular disease and acute or acute exacerbations of chronic diseases
	acute coronary syndrome (STEMI or NSTEMI)	-stable angina pectoris, old myocardial infarction, PCI or CABG for m in 6 months interval	ECG criteria of STEMI, angiographically proven coronary thrombi	≥50% stenosis in at least one main coronary artery	CAD confirmed by anamnestic data, results of daily ECG Holter monitoring, coronary angiography.
**Sample size**	CAD 19 Ctrl 19	CAD 39 Ctrl 30	CAD 22 Ctrl 20	CAD 186 Ctrl 123	CAD 29 Ctrl 30
**Technique**	16S rRNA Illumina MiSeq	T-RFLP	16S rRNA V3-V4 Illumina Miseq	16S rRNA V3-V4 Illumina MiSeq	16S rRNA V3-V4 Illumina MiSeq
**Study design**	Case-control	Cross-sectional	Case-control	Cross-sectional	Cross-sectional
**First author/year**	Alhmoud T., et al. [38] 2019	Emoto T., et al. [39] 2016	Kwun J., et al. [40] 2020	Zheng Y, et al. [41] 2020	Ivashkin, V., et al. [42] 2019
**RoB NOS**	7	9	8	9	7
**Diversity** **α β**	No data	No data	+	No data	No data
	No data	−	−	−	No data
**Major outcome-** **CAD relative abundance**	*Bacteroidetes ▼* *Lactobacillales ▲*	*Escherichia coli ▲* *R. gnavus ▲* *Bacteroides ▼* *Streptococcus ▲*	*Enterococcus ▲* *Lactobacillus ▲* *Bacteroides ▼* *Fusobacterium ▼* *Dorea ▼* *Streptococcus ▼*	*Bacteroides vulgatus ▼* *Bacteroides dorei ▼* *Faecalibacterium ▲ prausnitzii* *Prevotella copri ▲*	*Bacteroides ▼*
**Major exclusion criteria**	acute coronary syndrome systemic disease, including hepatic, renal, collagen disease and malignancy, antibiotic treatment	ongoing infectious diseases, cancer, renal, or hepatic failure, stroke, use of antibiotics within 1 month of sample collection.	kidney dialysis acute infection, gastrointestinal diseases cancer, treatments with antibiotics or probiotics within one month	Patients with: acute coronary syndrome, with systemic disease: including hepatic, renal, collagen disease and malignancy, antibiotics	heart failure, renal and hepatic disease, malignancies, inflammatory disease
**Major inclusion criteria** **CAD Control**	no history of coronary or another vascular disease, no symptoms indicating angina, no ischemic abnormality in ECG	asymptomatic, no history of CAD, renal failure, systemic disease, and stroke	No data	Patients with coronary risk factors: hypertension, diabetes, dyslipidemia Without a present or past history of coronary or other vascular diseases	Patients with coronary risk factors
	stable angina, old myocardial infarction, PCI or CABG at least 6 months interval, 75% stenosis of coronary artery	confirmed by coronary angiography, and ≥50% stenosis in single or multiple vessels	coronary angiography or coronary computed tomography angioplasty	stable angina, old myocardial infarction, PCI or CABG ≥ 6 months before the present study. >75% stenosis.	CAD confirmed by a coronary angiography
**Sample size**	CAD 39 Ctrl 50	CAD 218 Ctrl 187	CAD 67 Ctrl 17	CAD 30 Ctrl 30	CAD 11 Ctrl 10
**Technique**	T-RFLP	Shotgun sequen- cing	16S rRNA V4-V5 Illumina Miseq	16S rRNA V3-V4 Illumina MiSeq	16S rRNA V3-V4 Illumina MiSeq
**Study design**	Case-control	Cross-sectional	Cross-sectional	Cross-sectional	Cross-sectional
**First author/year**	Emoto T. et al. [43] 2016	Jie Z.,et al. [44] 2017	Liu Z., et al. [45] 2019	Yoshida N. et al. [46] 2018	Yoshida N., et al. [47] 2019
**RoB** **NOS**	10	10	7	9	
**Diversity** **α β**	+	+	No data	+	
	+	+	+	+	
**Major outcome-** **CAD relative abundance**	*Bacteroides ▼* *Escherichia ▼* *Desulfovibrio ▲* *Parabacteroides ▲* *Streptococcus ▲* *Lacobacillus ▲*	*Gamaaproteobacteria ▲* *Enterobacteriaceae ▲* *Prevotellaceae ▼* *Escherichia-Shigella ▲* *Fusobacterium ▲* *Streptococcus ▲* *Bacilli ▼* *Lactobacillus ▼* *Lactobacillales ▼* *Coriobacteriales ▲*	*Ruminococcus Gnavus ▲* *Lachnospiraceae ▼* *Ruminococcus* *Gauvreauii group ▼*	*Bacteroidetes ▼* *Bacteroidia ▼* *Bacilli ▲* *Gammaproteobacteria ▲* *Bacteroidales ▼* *Lactobacillales ▲*	
**Major exclusion criteria**	No history of unstable angina, myocardial infarction, stroke, cancers, coronary revascularization.	history of GIT surgery or organic disease, history of stroke, hypertension, diabetes, kidney disease infection within one the month of the study or the use of a probiotic, antacid, antibiotic	prior gastrointestinal surgery, the current administration of antibiotics or probiotics, history of IBD or auto-immune diseases,	history of acute or chronic intestinal disease, active cancer aged below 30 and over 80, history of acute coronary syndrome or typical angina (HC)	
**Major inclusion criteria** **CAD Control**	Without significant stenosis in coronary arteries	healthy volunteers from the hospital health examination center	abnormal peripheral endothelial dysfunction without CAD based on clinical history, non-invasive stress testing, and coronary imaging studies	randomly selected. sex and age-matched to CAD group, without CAD	
	SA and AMI criteria. The coronary angiography was performed on all patients.	coronary angiography, ECG changes	history of PCI or CABG, coronary arteries diagnosed by coronary angiography or computed tomography coronary angiography	aged 30–79, hospitalization 12–18 months before the evaluation for elective PCI ACS: STEMI, NSTEMI, UA	
**Sample size**	CAD 141 Ctrl 49	CAD 60 Ctrl 30	CAD 88 Ctrl 114	CAD 169 Ctrl 166	
**Technique**	16S rRNA Illumina HiSeq	Phusion High-Fidelity PCR Master Mix	16S rDNA IM-TORNADO	16S rRNA	
**Study design**	Cross-sectional	Cross-sectional	Cross-sectional	Cross-sectional	
**First author/year**	Dong Ch,, et al., 2021 preprint [48]	Gao J., et al. [49] 2020	Toya T, et al. [50] 2021	Sawicka- Śmiarowska et al. [27] 2021	
**RoB** **NOS**	10	10	10	10	
**Diversity** **α β**	+	No data	+	+	
	−	+	+	+	
**Major outcome-** **CAD relative abundance**	*Prevotellaceae ▲* *Fusobacterium ▼* *Bacteroides ▼* *Parabacteroides ▲*	*Lachnospiraceae ▼* *R. gnavus ▲*	*Firmicutes ▲* *Bacteroides ▼* *Bacteroidetes ▼* *Bacteroidia ▲*	*Bacteroides ▼* *Bacilli ▲* *Firmicutes ▼* *Gammaprotebacteria ▲* *Lachnospiracea ▼* *Escherichia-Shigella ▲* *Lactobacillus ▲* *Bacteroidia ▼* *Lactobacillales ▲* *Bacteroidales ▼* *Coriobacteriales ▲* *Christensellaceae ▼* *Streptococcus ▲*	
**Major exclusion criteria**	IBD, hepatitis B or cirrhosis, cancer, organ failure, exposure to probiotics or prebiotics within one month; receiving treatment with antibiotics,	gastrointestinal surgery, current administration of antibiotics and a probiotic, history of IBD and auto-immune diseases	probiotics, antibiotics within a month before sample gastrointestinal surgery;history of alcohol abuse, diabetes, gastrointestinal disease	cancer, infectious diseases: IBD, antibiotic or probiotic consumption within 1 month before sample collection	
**Major inclusion criteria** **CAD Control**	participants with no evidence of stenosis in the coronary artery	healthy volunteers, normal or <50% stenosis in coronary arteries	healthy volunteers	no history of CAD and other diseases from the exclusion criteria	
	coronary angiography, >50% stenosis	≥50% stenosis in at least one main coronary artery, patients with ACS	CAD confirmed by coronary angiography and CABG or PCI, residents of southern China, 50–85 years.	CAD confirmed by a coronary angiography	
**Sample size**	CAD 45 Ctrl 19	CAD 53 Ctrl 53	CAD 29 Ctrl 34	CAD 70 Ctrl 98	
**Technique**	16S rRNA V4 Ion Torrent	16S rDNA V3-V5 IM-Tornado	16S rRNA V3-V5 Illumina Miseq	16S rRNA V4 Illumina MiSeq	
**Study design**	Cross-sectional	Cross-sectional	Cross-sectional	Cross-sectional	
**First author/year**	Hu Jl, et al. [51] 2021	Toya T, et al. [52] 2020	Cui L., et al. [53] 2017	Zhu Q.et al. [54] 2018	
**RoB** **NOS**	9	7	7		
**Diversity** **α β**	+	No data	No data		
	+	+	No data		
**Major outcome-** **CAD relative abundance**	*Firmicutes ▲* *Bacteroides ▲* *Bacteroidetes ▼* *Gammaproteobacteria ▲* *Bacteroidia ▼* *Desulfovibrio ▲* *Prevotella ▼* *Bacteroidales ▼* *Christensellaceae ▲*	*Lachnospiraceae* *▼*	*Bacteroidetes ▼* *Bacteroidales ▼* *Coriobacteriales ▲*		
**Major exclusion criteria**	antibiotics, probiotics, or prebiotics for at least 3 months before sampling, acute and chronic inflammatory diseases, tumors	Prior gastrointestinal surgery, the current administration of antibiotics, IBD, malignancy, auto-immune disease	renal disease, malignancy, ongoing infectious disease, hepatic disease, use of antibiotics within four weeks before sample collection.		
**Major inclusion criteria** **CAD Control**	Tibetan native residents family members of the patients	Without CAD based on clinical history, non-invasive stress testing, and coronary imaging studies	healthy volunteers		
	Tibetan native residents coronary artery stenosis >50% ages 40–70 years	typical symptoms, the ECG pattern, cardiac enzyme raise, coronary angiography	CAD confirmed by coronary angiography, and patients with ≥50% stenosis in single or multiple vessels		
**Sample size**	CAD 18 Ctrl 23	CAD 19 Ctrl 25	CAD 15 Ctrl 15		
**Technique**	16S rRNA regions Illumina Hiseq	16S rRNA V3-V4 Illumina Miseq	16S rRNA V3-V4 Illumina Miseq		
**Study design**	Cross-sectional	Case-control study	Cross-sectional		
**First author/year**	Liu F. et al., 2020 [55]	Chiu et al., 2022 [56]	Choroszy et. al. [57] 2022		

### 3.3. Meta-Analysis Results

The gut microbiome profile in CAD patients and controls was compared using 16S rDNA gene sequencing in fecal samples. Although 16S rDNA can deliver the resolution required for taxonomic classification at species and strain levels, it generally involves sequencing the entire 16S rRNA gene. Since the study focused only on the V3-V4 region, the meta-analysis of species or lower taxonomic groups was not performed.

**Figure 3:**
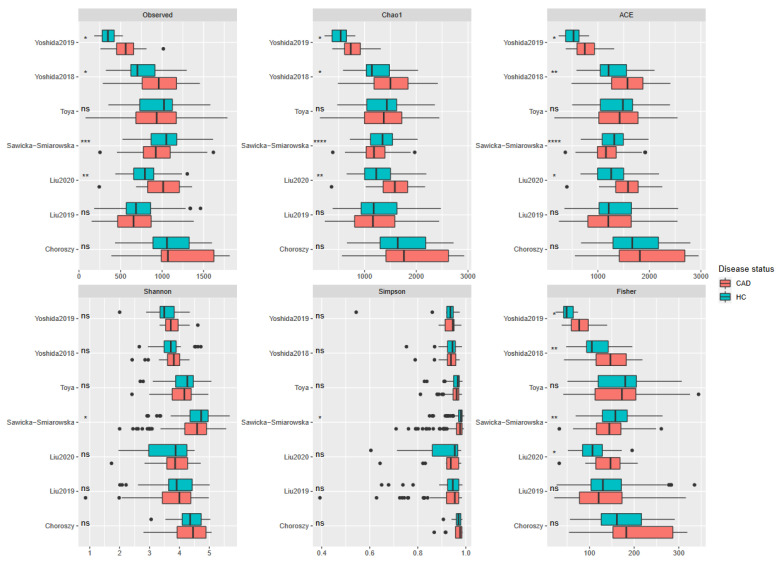
Alpha diversity results for individual studies. Asterixis describe statistically significant results: *-*p* ≤ 0.05, **-*p* ≤ 0.01, ***-*p* ≤ 0.001, ****-*p* ≤ 0.0001, ns-non-significant; dots denote outlier values.

**Figure 4:**
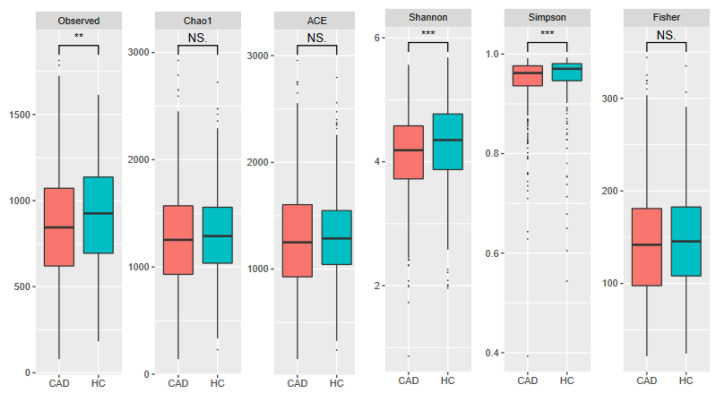
Alpha-diversity results for combined dataset. Asterixis describe statistically significant results: **-*p* ≤ 0.01, ***-*p* ≤ 0.001, ns-non-significant; dots denote outlier values.

**Figure 5:**
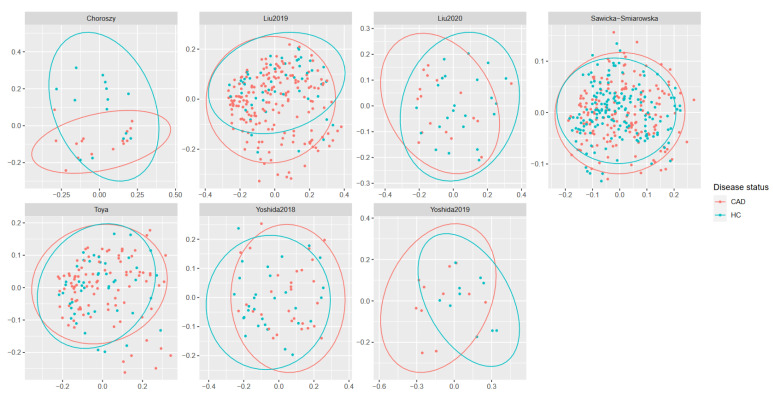
Beta-diversity measured by UniFrac.

Three of the evaluated alpha-diversity measures were decreased in CAD patients. These included Shannon (*p* = 0.00025) and Simpson indices (*p* < 0.0001), both of which measure evenness and observed OTUs (*p* = 0.0015), indicating richness. Other measures (Chao1, ACE, and Fisher indices) evaluated in this study did not significantly differ between groups (Figure 3 and Figure 4). All (except Toya et al. [50]) datasets showed significant differences in beta-diversity (measured with UniFrac) between controls and patients with CAD. However, PCoA plots (Figure 5) showed notable overlap between patients with CAD and healthy controls, without a clear boundary between samples.

**Figure 6:**
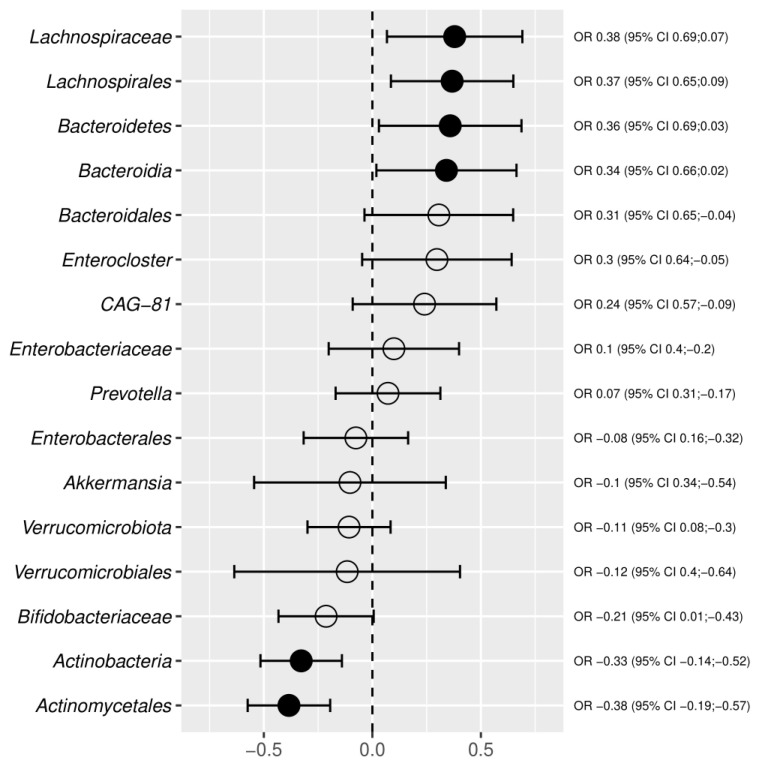
The results of random-effects meta-analysis for effect sizes based on the mean differences in centered log ratios. The left side of the graph describes bacteria with increased relative abundance in CAD, and the right side of the chart represents bacteria with decreased relative abundance in CAD. The filled dot denotes a statistically significant result of the random-effects meta-analysis.

**Figure 7:**
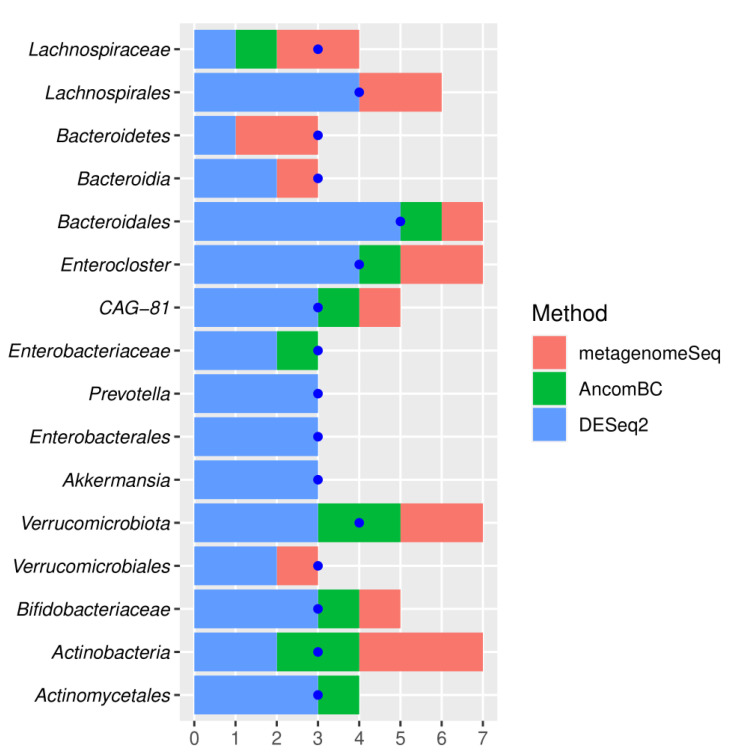
The results of a meta-analysis. Statistical significance testing for single datasets using metagenomeSeq, AncomBC, and DESeq2. The number of studies in which a given taxon was detected as significant by at least one method is denoted by a blue dot. Stacked bar charts show how many studies given differential abundance testing methods reported substantial results.

**Figure 8:**
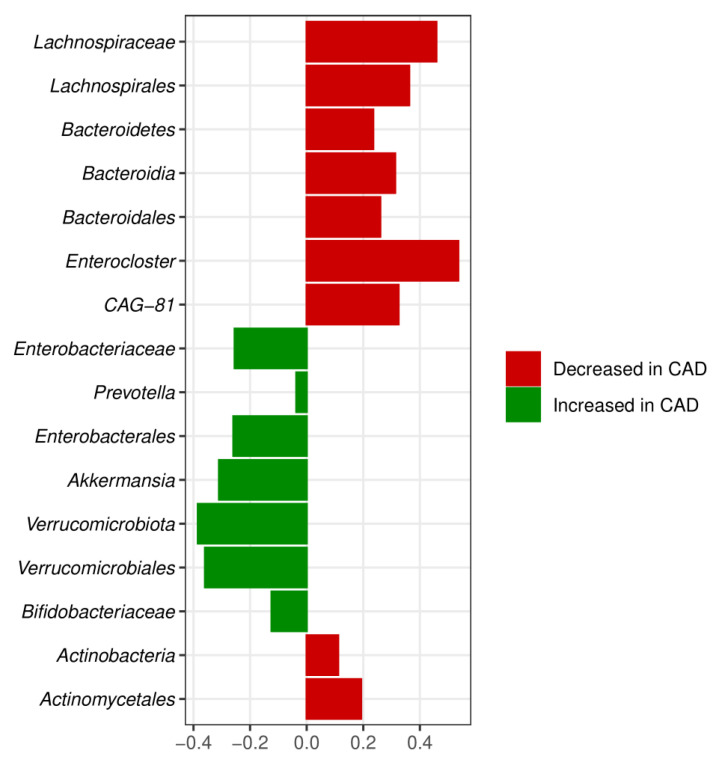
The results of a meta-analysis. Only bacteria detected as statistically significant metagenomeSeq, Ancom-BC, or DESeq2 in at least three of seven studies were included. Relative abundances (reported as log-fold change) for the pooled dataset.

The meta-analysis combined CAD with three phyla, i.e., *Bacteroidetes*, Actinobacteria, and Verrucomicrobiota (Figure 6, Figure 7 and Figure 8). The relative abundance of *Bacteroidetes* was decreased, whereas Actinobacteria and Verrucomicrobiota were increased in CAD compared to the control group. The *Bacteroidetes* depletion in CAD was also confirmed at the class, order, and genus levels (Figure 2) supporting the systematic review results. The Actinobacteria overrepresentation in CAD was further confirmed by the increased relative abundance of the order Actinomycetales and the family Bifidobacteriaceae.

Increased Verrucomicrobiota phylum in CAD versus the control group was also associated with increased Verrucomicrobiales order and Akkermansia genus. However, the confidence interval for Akkermansia significantly overlapped between the increase and decrease of abundance, indicating the low reliability of this result (Figure 6).

Additionally, the meta-analysis showed an increase in Proteobacteria at the level of Enterobacteriales and *Enterobacteriaceae* family. The alterations within the Firmicutes phylum concerned the *Lachnospiraceae* family and Enterocloster genus, which were decreased in CAD versus the control group. Additionally, the reduced abundance of the CAG-81 genus from a class of Clostridia was observed in CAD patients. Overall, the meta-analysis combined gut microbiota in CAD patients with main bacterial phyla, i.e., Proteobacteria, *Bacteroidetes*, Actinobacteria, Firmicutes, and Verrucomicrobiota, at different taxonomic ranks. The convergent results of systematic review and meta-analysis concerned *Enterobacteriaceae*, *Bacteroidetes*, and *Lachnospiraceae*. In contrast with the systematic review, the meta-analysis showed differences within the Verrucomicrobiota phylum in CAD patients versus the control group.

## 4. Discussion

The growing evidence indicates that altered gut microbiota plays a crucial role in coronary CAD as a risk factor for the disease’s outcome [58]. The gut microbiota in healthy individuals claims gut homeostasis, imparts resistance to the colonization of new species, and maintains a symbiotic relationship with the host. On the contrary, the shift in the diversity and abundance of gut microbiota facilitates the overgrowth of potentially pathogenic bacteria causing inflammatory processes and the evolution of various diseases [59]. An imbalance in the quantity of specific bacterial taxa in the gut microbiota correlates with a deficiency or excess of bacterial metabolites that fundamentally affect the physiological status of the host cells, including endothelial cells. Depending on the concentration, bacterial metabolites, e.g., trimethylamine-N-oxide (TMAO), lipopolysaccharide (LPS), or indoxyl sulfate, may yield direct toxic effects on the endothelium or indirect toxicity through their modulatory effects on hormones and biologically active compounds of the host organism [60].

The gut microbiota is dominated by five bacterial phyla, namely *Bacteroidetes*, Firmicutes, Actinobacteria, Proteobacteria, and Verrucomicrobiota, with Firmicutes and *Bacteroidetes* accounting for more than 90% of the overall gut microbiome [61]. Interestingly, the most common alterations reported in CAD are related to decreased *Bacteroidetes* and increased Firmicutes abundance [52].

The systematic review and meta-analysis performed in the study confirmed a significant decrease in the *Bacteroidetes* taxa in CAD. The decrease of *Bacteroidetes* in the gut microbiota carries profound health implications. These Gram-negative obligate anaerobic bacteria have several beneficial effects on the human body and play an essential role in maintaining a healthy gut ecosystem [52]. First, *Bacteroidetes* is involved in the degradation of non-digestible dietary carbohydrates and host-derived carbohydrates from gastrointestinal tract secretions yielding butyrate and acetate, which can lower serum lipid levels by blocking cholesterol synthesis [62,63]. Moreover, the *Bacteroidetes* taxa esterify absorbable cholesterol to coprostanol, a nonabsorbable sterol excreted in feces, thus lowering the blood level of cholesterol [62]. Notably, the high efficiency of cholesterol to coprostanol metabolism is suggested to reduce the risk of CAD [64]. Second, the *Bacteroidetes* capsular polysaccharide antigen (PSA) is vital in activating the T-cell-dependent immune response that can affect the development and homeostasis of the host immune system [65,66]. PSA of Bacteroides promotes CD4+ T cell differentiation, the balance of Th1 and Th2 populations, and the differentiation of regulatory T cells (Treg) [67,68]. On an atherosclerotic-prone mice model, Yoshida et al. [46] demonstrated that mice supplementation with Bacteroides ameliorated endotoxemia, reduced TLR4 expression and activation, and lowered plasma levels of pro-atherogenic cytokines such as IL-2, IL-4, IL-6, IL-17A, INF-γ, and TNF-α. Third, some Bacteroides species directly impact microbial LPS synthesis in the human gut, lowering systemic endotoxemia involved in the onset and progression of atherosclerosis. Penta- and tetra-acylated lipids A in LPS of Bacteroides are structurally distinct from the hexa-acylated LPS of E. coli and, in contrast with E. coli LPS, elicit reduced TLR4 response. TLR4 expression increases after LPS exposure in a dose-dependent manner, and TLR-mediated dendritic cell activation and maturation upregulates the histocompatibility complex, costimulatory molecules, cytokine production, and T cell activation. The quantity of Bacteroides tends to be negatively correlated with fecal LPS levels [46,68]. Arumugam et al. [69] demonstrated a higher incidence of symptomatic atherosclerosis in individuals with decreased Bacteroides abundance. Hence, reduced Bacteroides in the gut microbiome seem to be an important factor predisposing to CAD.

The current meta-analysis demonstrated decreased relative abundance of the *Lachnospiraceae* family of Firmicutes phylum in CAD versus the control group, which is in concordance with other studies [50,54]. *Lachnospiraceae*, similarly to *Bacteroidetes* taxa, produce butyrate and reduce cholesterol to coprostanol, lowering blood cholesterol levels [70]. Therefore, the *Lachnospiraceae* decrease in CAD may amplify the adverse effects of Bacteroides depletion of the gut microbiota. Toya et al. demonstrated that advanced CAD patients had a reduced relative abundance of *Lachnospiraceae* NK4B4 and other *Lachnospiraceae* family members, which may suggest a possibility that butyrate depletion could lead to an increase in inflammation. Moreover, the meta-analysis demonstrated in CAD patients decreased Enterocloster genus, comprising re-classified Clostridium species.

According to the systematic review, CAD was associated with an increased abundance of Enterobacteria, *Lactobacillus*, and Coriobacterium taxa. *Lactobacillus* is a well-known lactic acid producer with beneficial effects on human health, as demonstrated in several studies [71,72]. However, none of these studies evaluated current *Lactobacillus* levels before supplementation with these probiotics. Hence, there is a possibility that the overgrowth of specific *Lactobacillus* strains in gut microbiota may have adverse effects on human health. According to Ferrarese et al. [73], some probiotic strains such as L.acidophilus, L.ingluviei, L. fermentum, and L. delbrueckii are linked to a paradoxical significant weight-gain effect both in animal and human studies. According to Quiepo-Ortuno’s [74] study on an animal model, leptin and ghrelin, energy metabolism hormones, seem to be regulated by lactic acid-producing bacteria. Their study demonstrated a positive correlation between the abundance of Bifidobacterium and *Lactobacillus* and serum leptin levels and a significant negative correlation between the number of Clostridium, Bacteroides, and Prevotella and serum leptin levels.

Leptin is a hormone produced primarily by adipose cells and enterocytes that helps regulate energy balance by inhibiting hunger, which diminishes fat storage in adipocytes. However, obesity promotes insulin resistance and increases serum insulin levels, and high insulin levels increase leptin levels, eventually leading to leptin resistance in the nervous system and adipose tissue. This causes leptin to not reduce food intake and body weight in obese individuals [75]. Therefore, an increased abundance of *Lactobacillus* and Bifidobacterium and a decreased abundance of Bacteroides taxa in obese individuals may result in leptin resistance that fuels obesity, a well-known risk factor of CAD. Whether the increase in *Lactobacillus* is related to leptin levels in humans needs to be determined.

Moreover, it has been shown that the therapeutic effects of statins are attenuated by the abundance of *Lactobacillus* and Bifidobacterium, which renders these drugs relatively ineffective in decreasing LDL, confirming the putative role of lactic acid bacteria in leptin resistance [76]. Puurunen et al. [77] demonstrated that high plasma leptin levels predict the short-term occurrence of congestive heart failure, cardiac death, and acute coronary syndrome in patients with CAD independently of established risk factors.

On the contrary, ghrelin is a hormone produced by enteroendocrine cells in the gastrointestinal tract that stimulates food intake, fat deposition, and growth hormone release. Moreover, ghrelin and its receptor GHS-R1a have a cardioprotective effect on the cardiovascular system via the modulation of sympathetic activity and hypertension, enhancement of vascular activity and angiogenesis, inhibition of arrhythmias, reduction in heart failure, and inhibition of cardiac remodeling after myocardial infarction [78]. Interestingly, a meta-analysis aimed to summarize the available data regarding the circulating levels of ghrelin in patients with CAD published by Niknam et al. [79] also combined significantly lower circulating ghrelin levels with CAD. According to Torres-Fuentes et al. [80], lactate produced by *Lactobacillus* and Bifidobacterium attenuates ghrelin-mediated signaling through the GHSR-1a. On an animal model, Queipo-Ortuño et al. [74] demonstrated that serum ghrelin levels were negatively correlated with Bifidobacterium and *Lactobacillus* and positively correlated with Bacteroides and Prevotella. These data strongly suggest that an overabundance of lactic acid bacteria in the gut microbiome of patients with CAD may negatively impact patients’ energy metabolism leading to weight gain and obesity. Notably, the abundance of *Lactobacillus* in the gut microbiome is regulated by proatherogenic TMAO, a well-known predictor of cardiovascular disease. Hoyles et al. [81] demonstrated that TMAO stimulates the growth of lactic acid bacteria and lactate production. The level of TMAO, in turn, depends on the abundance of bacterial taxa producing TMAO precursor, i.e., trimethylamine (TMA) from dietary choline, mainly Gamma- and Betaproteobacteria, and some Firmicutes [82]. Interestingly, TMAO also stimulates the growth of *Enterobacteriaceae* and may account for the increased abundance of *Enterobacteriaceae* and *Lactobacillus* taxa in CAD [81]. Additionally, increased Enterobacteria correlates with increased fecal indole and serum indoxyl sulfate, a cardiotoxic uremic toxin [83]. The increase in the plasma levels of these uremic toxins is reported to accelerate the development of atherosclerotic plaque [21]. Moreover, an increased abundance of Gram-negative *Enterobacteriaceae* taxa may be an essential source of endotoxin. In gut dysbiosis, the endotoxin leaks into the circulation, inducing inflammation and accelerating CAD progression [57].

Jie et al. [44] performed a metagenomic shotgun-sequencing on stool samples from 218 patients with atherosclerotic CVDs and 187 healthy controls and identified, apart from *Enterobacteriaceae*, an increased abundance of *Streptococcus* spp. in atherosclerotic cardiovascular disease which is in concordance with the current study. The link between the gut *Streptococcus* species with atherosclerosis has been demonstrated in the current study and is well-established in other studies [44,84,85]. Hashizume-Takizawa et al. [84] on the hyperlipidemic mouse model demonstrated that atherosclerotic plaque formation increased significantly in the S. sanguis-challenged mice compared to the control group. Moreover, challenged mice showed increased expression levels of mRNAs of proinflammatory cytokines in the aorta and atherosclerosis-related mediators in the blood. The role of 73 *Streptococcus* species in aortic inflammation and atherosclerosis progression has been confirmed recently by computed tomography-derived coronary artery calcium score in a large cohort of middle-aged Swedes and validated in a geographically separate case-control study of symptomatic atherosclerotic disease. The study supported that gut *Streptococcus* spp. were independently associated with endogenous and exogenous atherogenic plasma metabolites, inflammatory and infection markers, and bacterial homologs in the oral cavity, which were associated with worse oral health [86].

The Coriobacteriales order, which according to the systematic review, was more abundant in CAD patients than controls, includes pathobionts of human gut microbiota. With Collinsella as its dominant taxon, these bacteria can affect host metabolism by altering intestinal cholesterol absorption, decreasing glycogenesis in the liver, and increasing triglyceride synthesis. Karlsson et al. [86], using shotgun sequencing of the gut metagenome, demonstrated increased Collinsella genus in patients with stenotic atherosclerotic plaques in the carotid artery. According to a study on an animal model, the presence of Corriobacteriaceae in the mouse gut correlated with decreased hepatic glycogen and glucose levels, enhanced triglyceride synthesis, and the activity of Cyp3a11, a hepatic detoxification enzyme [87]. Lahti et al. [88] identified a positive correlation between the abundance of human serum cholesterol and the genus Collinsella. According to biochemical lipid analysis, the Collinsella genus explicitly correlates with total cholesterol and LDL but not HDL, supporting data generated in studies on animal models [86,89].

There are several limitations to our study. First, most studies included in our systematic review were based on 16s rRNA sequencing focused on the V3-V4 regions, which can barely classify microbiota to the strain level. Specific metabolites of bacterial strains may exert pronounced effects on gut homeostasis. Hence, accurate characterization of the gut microbiota at the species level may be of great importance concerning preventing CAD by regulating the abundance of bacterial taxa.

Another limitation is the difference in characteristics of the studied populations in the included studies. Several studies have shown significant differences in age, gender distribution, BMI, and diabetes mellitus frequency, which are also risk factors for CAD. All these factors are also associated with changes in the gut microbiome [90,91,92,93]. Hence, it can be hypothesized that these factors, but not CAD itself, cause the microbiome alterations detected in our study. However, since most of the included studies did not show significant differences in the frequency of these risk factors, we consider this hypothesis unlikely.

The meta-analysis and systematic review pointed out differences in the gut microbiota composition in CAD patients compared to healthy controls. One of the most striking differences was the decrease in the beneficial Bacteroides and Lachnospira combined with the increase in enterobacteria, Actinobacteria, and Verrucomicrobiota in CAD patients. These alterations in the gut microbiota composition are associated with quantitative changes in atherogenic bacterial metabolites, e.g., LPS, TMAO, and uremic toxins, that increase the risk of developing or progressing CAD.

The gut microbiota composition, however, can be modulated by an appropriate diet. Therefore, knowledge and understanding of the role of the gut microbiota in CAD pathomechanism are crucial in preventing the coronary artery disease and slowing its progression. Targeting personalized medicine with dietary selection or supplementation with beneficial bacterial species could help reduce cardiovascular morbidity and mortality. However, before this can happen, an in-depth understanding of how the gut microflora changes under the diet and impacts mutual interactions with the host organism is imperative.

## Data Availability

The part of data that support the findings of this study are openly available under the accession numbers: PRJDB7456, PRJDB6472, PRJNA550301, PRJNA503710.

## Supplementary Materials

The following supporting information can be downloaded at: https://www.mdpi.com/article/10.3390/metabo12121165/s1. S1: Search strategy; Table S2: PRISMA 2020 for Abstracts Checklist; S3: PRISMA 2020 Checklist; S4: The Newcastle-Ottawa Assessment Quality Scale; Table S5: Characteristics of studies included in the meta-analysis; Table S6: Characteristics of participants included studies.
