# Supplementary material for: Human Gut Microbiota in Coronary Artery Disease: A Systematic Review and Meta-Analysis
Source: Metabolites. 2022 Nov 23;12(12):1165. doi: 10.3390/metabo12121165 (PMC9788186; doi:10.3390/metabo12121165)
Supplement: Supplementary file 1 [file metabolites-12-01165-s001.zip › metabolites-2010784-supplementary.pdf]

Human Gut Microbiota in Coronary Artery Disease: A Systematic Review and Meta-Analysis.

Supplementary materials

S1 PRISMA 2020 for Abstracts Checklist

Table S2 PRISMA 2020 Checklist

S3 Search strategy

S4: The Newcastle-Ottawa Assessment Quality Scale

Table S5 Characteristics of studies included in the meta-analysis

Table S6 Characteristics of participants in included studies

S1. PRISMA 2020 for Abstract checklist.

Section and Topic	Item #	Checklist item	Reported (Yes/No)
**TITLE**			
Title	1	Identify the report as a systematic review.	Yes
**BACKGROUND**			
Objectives	2	Provide an explicit statement of the main objective(s) or question(s) the review addresses.	Yes
**METHODS**			
Eligibility criteria	3	Specify the inclusion and exclusion criteria for the review.	Yes
Information sources	4	Specify the information sources (e.g. databases, registers) used to identify studies and the date when each was last searched.	Yes
Risk of bias	5	Specify the methods used to assess risk of bias in the included studies.	Yes
Synthesis of results	6	Specify the methods used to present and synthesise results.	Yes
**RESULTS**			
Included studies	7	Give the total number of included studies and participants and summarise relevant characteristics of studies.	Yes
Synthesis of results	8	Present results for main outcomes, preferably indicating the number of included studies and participants for each. If meta-analysis was done, report the summary estimate and confidence/credible interval. If comparing groups, indicate the direction of the effect (i.e. which group is favoured).	Yes
**DISCUSSION**			
Limitations of evidence	9	Provide a brief summary of the limitations of the evidence included in the review (e.g. study risk of bias, inconsistency and imprecision).	Yes
Interpretation	10	Provide a general interpretation of the results and important implications.	Yes
**OTHER**			
Funding	11	Specify the primary source of funding for the review.	Yes
Registration	12	Provide the register name and registration number.	Yes

Table S2. PRISMA 2020 Checklist

Section and Topic	Item #	Checklist item	Location where item is reported
**TITLE**			
Title	1	Identify the report as a systematic review.	Page 1
**ABSTRACT**			
Abstract	2	See the PRISMA 2020 for Abstracts checklist.	Page 2-3
**INTRODUCTION**			
Rationale	3	Describe the rationale for the review in the context of existing knowledge.	Page 4
Objectives	4	Provide an explicit statement of the objective(s) or question(s) the review addresses.	Page 4
**METHODS**			
Eligibility criteria	5	Specify the inclusion and exclusion criteria for the review and how studies were grouped for the syntheses.	Page 5-6
Information sources	6	Specify all databases, registers, websites, organisations, reference lists and other sources searched or consulted to identify studies. Specify the date when each source was last searched or consulted.	Page 5
Search strategy	7	Present the full search strategies for all databases, registers and websites, including any filters and limits used.	Page 5 and supplementary materials
Selection process	8	Specify the methods used to decide whether a study met the inclusion criteria of the review, including how many reviewers screened each record and each report retrieved, whether they worked independently, and if applicable, details of automation tools used in the process.	Page 6
Data collection process	9	Specify the methods used to collect data from reports, including how many reviewers collected data from each report, whether they worked independently, any processes for obtaining or confirming data from study investigators, and if applicable, details of automation tools used in the process.	Page 6
Data items	10a	List and define all outcomes for which data were sought. Specify whether all results that were compatible with each outcome domain in each study were sought (e.g. for all measures, time points, analyses), and if not, the methods used to decide which results to collect.	Page 4-5
	10b	List and define all other variables for which data were sought (e.g. participant and intervention characteristics, funding sources). Describe any assumptions made about any missing or unclear information.	Page 4-5, Table 1
Study risk of bias assessment	11	Specify the methods used to assess risk of bias in the included studies, including details of the tool(s) used, how many reviewers assessed each study and whether they worked independently, and if applicable, details of automation tools used in the process.	Page 6 and supplementary materials
Effect measures	12	Specify for each outcome the effect measure(s) (e.g. risk ratio, mean difference) used in the synthesis or presentation of results.	Page 4-5
Synthesis methods	13a	Describe the processes used to decide which studies were eligible for each synthesis (e.g. tabulating the study intervention characteristics and comparing against the planned groups for each synthesis (item #5)).	Page 6
	13b	Describe any methods required to prepare the data for presentation or synthesis, such as handling of missing summary statistics, or data conversions.	Pages 6, 12
	13c	Describe any methods used to tabulate or visually display results of individual studies and syntheses.	Pages 6, 12
	13d	Describe any methods used to synthesize results and provide a rationale for the choice(s). If meta-analysis was performed, describe the model(s), method(s) to identify the presence and extent of statistical heterogeneity, and software package(s) used.	Pages 6, 12

Section and Topic	Item #	Checklist item	Location where item is reported
	13e	Describe any methods used to explore possible causes of heterogeneity among study results (e.g. subgroup analysis, meta-regression).	NA
	13f	Describe any sensitivity analyses conducted to assess robustness of the synthesized results.	NA
Reporting bias assessment	14	Describe any methods used to assess risk of bias due to missing results in a synthesis (arising from reporting biases).	NA
Certainty assessment	15	Describe any methods used to assess certainty (or confidence) in the body of evidence for an outcome.	NA
**RESULTS**			
Study selection	16a	Describe the results of the search and selection process, from the number of records identified in the search to the number of studies included in the review, ideally using a flow diagram.	Page 13
	16b	Cite studies that might appear to meet the inclusion criteria, but which were excluded, and explain why they were excluded.	
Study characteristics	17	Cite each included study and present its characteristics.	Page 8-11
Risk of bias in studies	18	Present assessments of risk of bias for each included study.	Supplementary materials
Results of individual studies	19	For all outcomes, present, for each study: (a) summary statistics for each group (where appropriate) and (b) an effect estimate and its precision (e.g. confidence/credible interval), ideally using structured tables or plots.	Figure 2
Results of syntheses	20a	For each synthesis, briefly summarise the characteristics and risk of bias among contributing studies.	NA
	20b	Present results of all statistical syntheses conducted. If meta-analysis was done, present for each the summary estimate and its precision (e.g. confidence/credible interval) and measures of statistical heterogeneity. If comparing groups, describe the direction of the effect.	Fig 3, supplementary materials
	20c	Present results of all investigations of possible causes of heterogeneity among study results.	NA
	20d	Present results of all sensitivity analyses conducted to assess the robustness of the synthesized results.	NA
Reporting biases	21	Present assessments of risk of bias due to missing results (arising from reporting biases) for each synthesis assessed.	NA
Certainty of evidence	22	Present assessments of certainty (or confidence) in the body of evidence for each outcome assessed.	NA
**DISCUSSION**			
Discussion	23a	Provide a general interpretation of the results in the context of other evidence.	Page 19-21
	23b	Discuss any limitations of the evidence included in the review.	Page 22-23
	23c	Discuss any limitations of the review processes used.	Page 22-23
	23d	Discuss implications of the results for practice, policy, and future research.	Page 21-22
**OTHER INFORMATION**			
Registration and protocol	24a	Provide registration information for the review, including register name and registration number, or state that the review was not registered.	Page 4
	24b	Indicate where the review protocol can be accessed, or state that a protocol was not prepared.	Page 4
	24c	Describe and explain any amendments to information provided at registration or in the protocol.	NA

Section and Topic	Item #	Checklist item	Location where item is reported
Support	25	Describe sources of financial or non-financial support for the review, and the role of the funders or sponsors in the review.	Page 22
Competing interests	26	Declare any competing interests of review authors.	[to trzeba bedzie potem dodac]
Availability of data, code and other materials	27	Report which of the following are publicly available and where they can be found: template data collection forms; data extracted from included studies; data used for all analyses; analytic code; any other materials used in the review.	NA

S3. Search terms

PubMed

(Human Microbiomes OR Human Microbiome OR Human Microflora OR Microbial Community Structure OR Human Flora OR Composition, Microbial Community OR Gut Microbiome OR Gut Flora OR Dysbiosis) AND (atherosclerosis or atherogenesis or Coronary atherosclerosis or Coronary Artery Disease or Coronary Atherosclerosis) NOT Review

Scopus:

("Human Microbiomes" OR "Human Microbiome" OR "Human Microflora" OR "Microbial Community Structure" OR "Human Flora OR Composition" OR "Gut Microbiome" OR "Gut Flora" OR "dysbiois") AND (atherosclerosis or atherogenesis or "Coronary atherosclerosis" or "Coronary Artery Disease" or "Coronary Atherosclerosis") AND NOT Review

Web of Science:

(TS=((Human Microbiomes OR Human Microbiome OR Human Microflora OR Microbial Community Structure OR Human Flora OR Composition, Microbial Community OR Gut Microbiome OR Gut Flora OR Dysbiosis) AND (atherosclerosis or atherogenesis or Coronary atherosclerosis or Coronary Artery Disease or Coronary Atherosclerosis)) NOT DT=(Review))

EMBASE:

('human microbiomes' OR 'human microbiome' OR 'human microflora' OR 'microbial community structure' OR 'human flora or composition' OR 'gut microbiome' OR 'gut flora' OR 'dysbiois') AND (atherosclerosis OR atherogenesis OR 'coronary artery disease' OR 'coronary atherosclerosis') NOT review

COCHRANE:

("Human Microbiome" OR "Human Flora" OR "Intestinal flora" OR "Gut Flora" OR "Gut Microbiota" OR "Gut Microbiota") AND ("Coronary Artery Disease" OR "Coronary Heart Disease" OR atherosclerosis OR atherogenesis OR "Coronary Atherosclerosis")

Clinical Trials:

(Human Microbiomes OR Human Microbiome OR Human Microflora OR Microbial Community Structure OR Human Flora OR Composition, Microbial Community OR Gut Microbiome OR Gut Flora OR Dysbiosis) | ("Coronary Artery Disease" OR "Coronary Heart Disease" OR atherosclerosis OR atherogenesis OR "Coronary Atherosclerosis")

S4. NEWCASTLE - OTTAWA QUALITY ASSESSMENT SCALE
(adapted for cross sectional studies)

Selection: (Maximum 5 stars)

1) Representativeness of the sample:
    a) Truly representative of the average in the target population. * (all subjects or random sampling)
    b) Somewhat representative of the average in the target population. * (non-random sampling)
    c) Selected group of users.
    d) No description of the sampling strategy.
2) Sample size:
    a) Justified and satisfactory. *
    b) Not justified.
3) Where there deviations from collected material and results unbalanced and likely to have affected the outcome?:
    a) Comparability between collected material and outcome is satisfactory. *
    b) Comparability between collected material and outcome is unsatisfactory.
    c) No description of the comparability between collected material and outcome.
4) Ascertainment of the exposure (risk factor):
    a) Validated measurement tool. **
    b) Non-validated measurement tool, but the tool is available or described.*
    c) No description of the measurement tool.

Comparability: (Maximum 2 stars)

1) The subjects in different outcome groups are comparable, based on the study designor analysis. Confounding factors are controlled.
    a) The study controls for the most important factor (select one). *
    b) The study control for any additional factor. *

Outcome: (Maximum 3 stars)

1)Assessment of the outcome:

    a) Independent blind assessment. **
    b) Record linkage. **
    c) Self report. *
    d) No description.
2) Statistical test:
    a) The statistical test used to analyze the data is clearly described and appropriate, and the measurement of the association is presented, includingconfidence intervals and the probability level (p value). *
    b) The statistical test is not appropriate, not described or incomplete.

This scale has been adapted from the Newcastle-Ottawa Quality Assessment Scale for cohort studies to perform a quality assessment of cross-sectional studies for the systematic review. The modification has been performed using modified scale available in the article Herzog, R., Álvarez-Pasquin, M.J., Díaz, C. *et al.* Are healthcare workers' intentions to vaccinate related to their knowledge, beliefs and attitudes? a systematic review. *BMC Public Health* 13**,** 154 (2013). https://doi.org/10.1186/1471-2458-13-154

Figure 1. Case-control study- the risk of bias

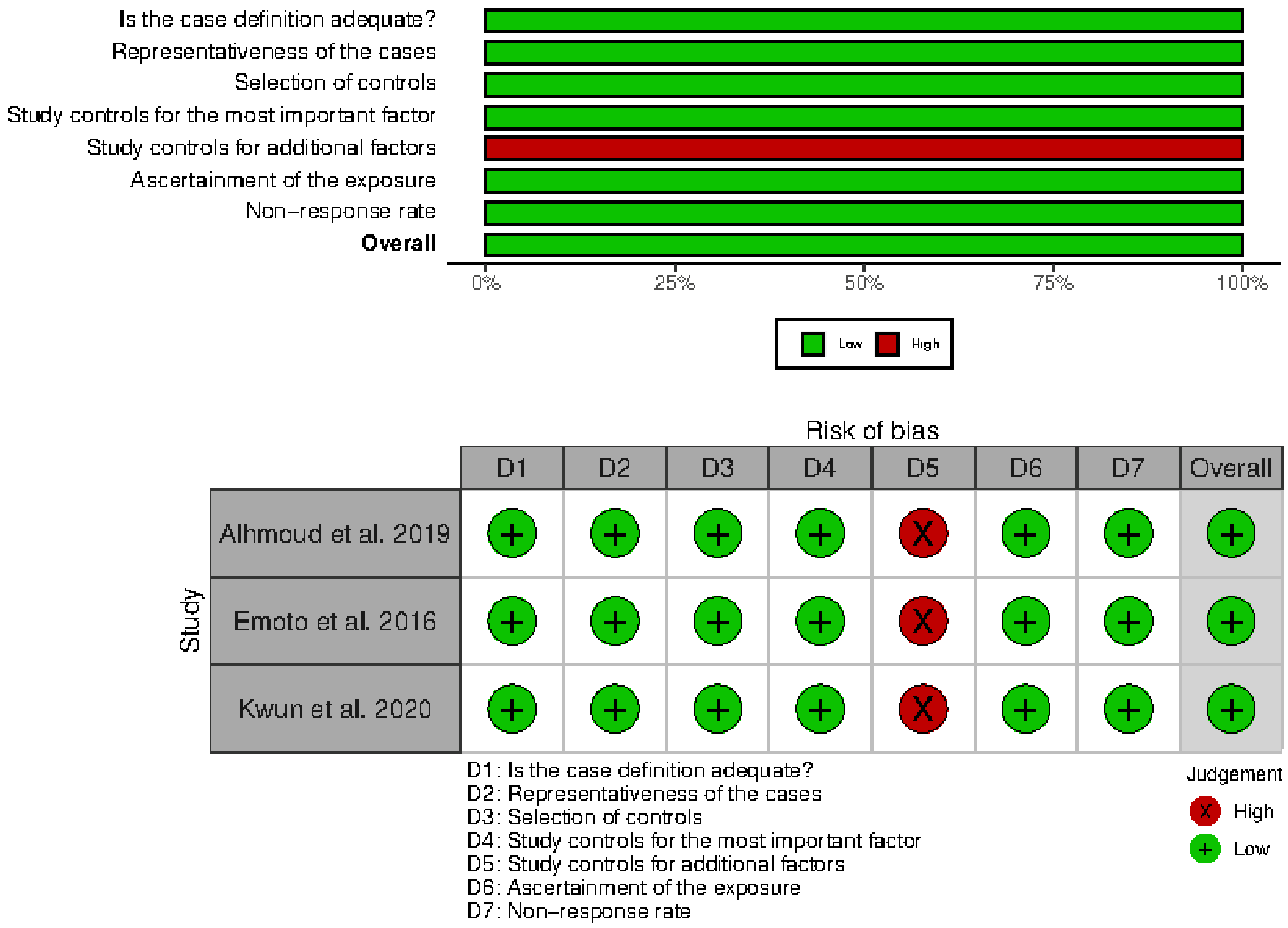

Figure 2 Cross-sectional study- the risk of bias

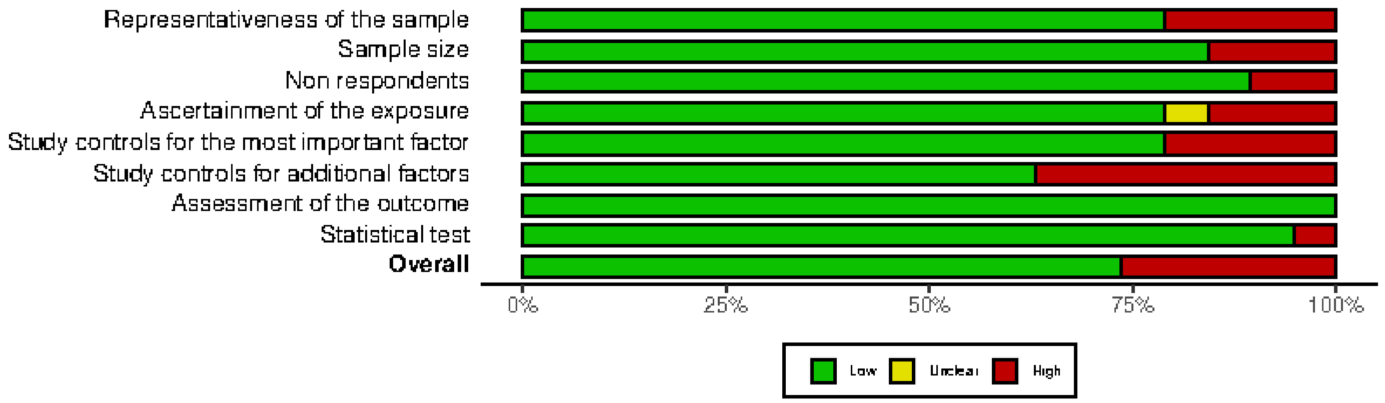

Figure 3. Cross-sectional study risk of bias

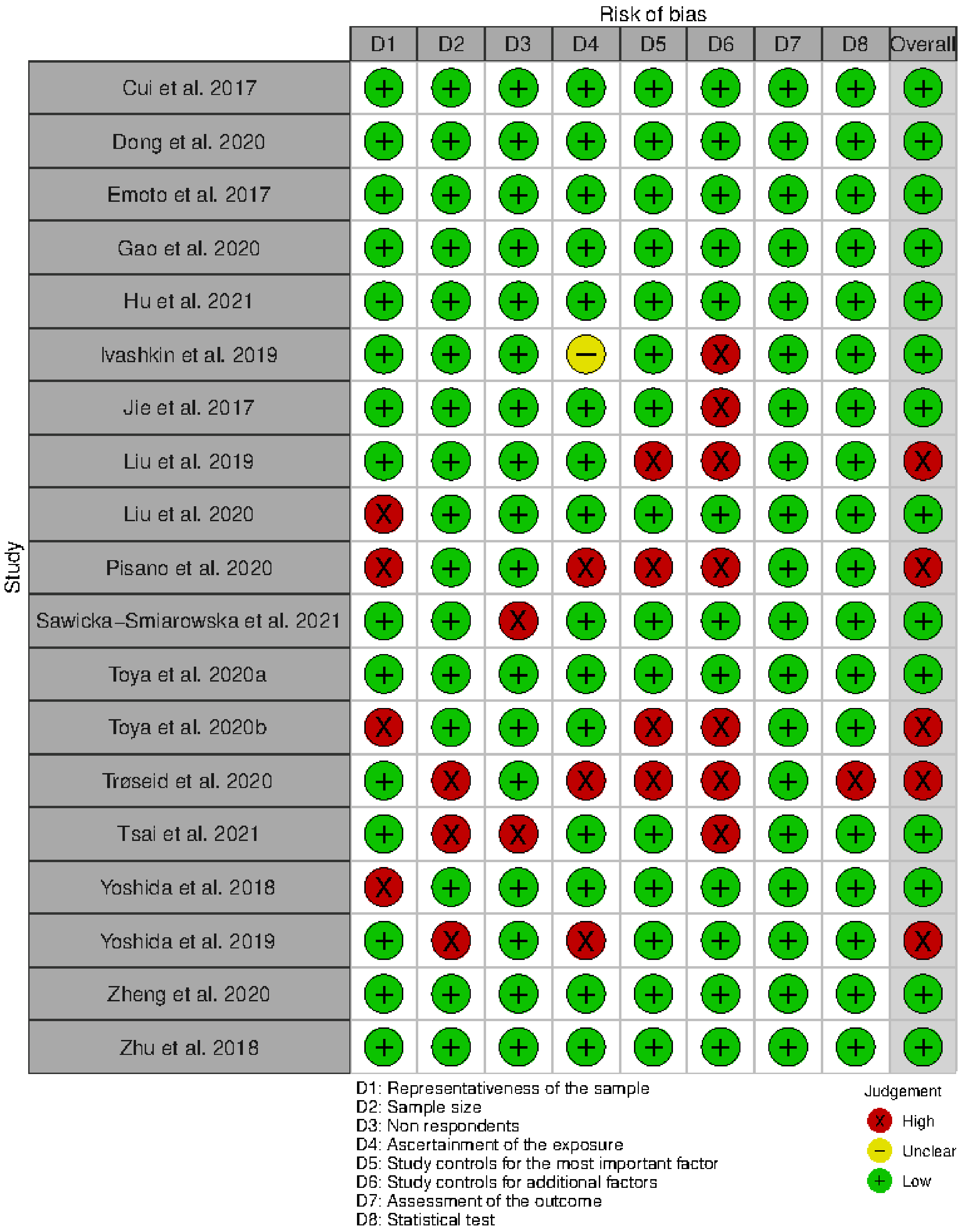

Table S5. Characteristics of studies included in the meta-analysis

Study	primer_F	primer_R	type	Bioproject	N patients (HC/CAD)
Choroszy et al. [1]	CCTACGGGAGGCTGCAG	GACTACAGGGGTATCTAATCC	16s		14/15
Yoshida et al. [2]	CTACGGGGGGCAGCAG	GGACTACNNGGGTATCTAAT	16s	PRJDB7456	10/11
Yoshida et al. [3]	CCTACGGGAGGCTGCAG	GACTACAGGGGTATCTAATCC	16s	PRJDB6472	30/30
Liu et al. [4]	CCTACGGGAGGCTGCAG	GGACTACNNGGGTATCTAAT	16s	PRJNA550301	23/18
Liu et al. [5]	CCTACGGGAGGCTGCAG	GGACTACNNGGGTATCTAAT	16s	PRJNA503710	52/186
Toya et al [6]	CCTACGGGAGGCAGCAG	CCGTCAATTCMTTTRAGT	16s		47/95
Sawicka-Śmiarowska [7]	GTGCCAGCMGCCGCGGTAA	GGACTACHVGGGTWTCTAAT	16s		166/169

[1] Choroszy, M., Sobieszczańska, B., Litwinowicz, K., et al.(2022). Co-toxicity of Endotoxin and Indoxyl Sulfate, Gut-Derived Bacterial Metabolites, to Vascular Endothelial Cells in Coronary Arterial Disease Accompanied by Gut Dysbiosis. *Nutrients*, *14*(3), 424. doi:10.3390/nu14030424

[2] Yoshida N, Sasaki K, Sasaki D, et al. Effect of Resistant Starch on the Gut Microbiota and Its Metabolites in Patients with Coronary Artery Disease. *J Atheroscler Thromb*. 2019;26(8):705-719. doi:10.5551/jat.47415

[3] Yoshida, N., Emoto, T., Yamashita, T., et al.(2018). Bacteroides vulgatus and Bacteroides dorei Reduce Gut Microbial Lipopolysaccharide Production and Inhibit Atherosclerosis. *Circulation*, *138*(22), 2486–2498. doi:10.1161/CIRCULATIONAHA.118.033714

[4] Liu, Fengyun et al. "Alterations of Gut Microbiome in Tibetan Patients With Coronary Heart Disease." *Frontiers in cellular and infection microbiology* vol. 10 373. 23 Jul. 2020, https://doi.org/10.3389/fcimb.2020.00373

[5] Liu H, Chen X, Hu X, et al. Alterations in the gut microbiome and metabolism with coronary artery disease severity. *Microbiome*. 2019;7(1):68. Published 2019 Apr 26. doi:10.1186/s40168-019-0683-9

[6] Toya T, Ozcan I, Corban MT, et al. Compositional change of gut microbiome and osteocalcin expressing endothelial progenitor cells in patients with coronary artery disease. *PLoS One*. 2021;16(3):e0249187. Published 2021 Mar 25. doi:10.1371/journal.pone.0249187

[7] Sawicka-Smiarowska E, Bondarczuk K, Bauer W, et al. Gut Microbiome in Chronic Coronary Syndrome Patients. *J Clin Med*. 2021;10(21):5074. Published 2021 Oct 29. doi:10.3390/jcm10215074

Table S6. Characteristics of participants in included studies

Author	Age			Gender, Male (n,%)			BMI			DM2		
	CAD	Ctrl	p-value	CAD	Ctrl	p-value	CAD	Ctrl	p-value	CAD	Ctrl	p-value
[1]	54.4 ± 2.2	49 ± 1.6	0.07	14 (74%)	13 (68%)	0.7	28.31 ± 1.9	24.3 ± 0.39	0.004	4 (21%)	0	N/A
[2]	61.1 ± 9.4	62.1 ± 6.4	P>0.05	33 (85%)	23 (77%)	P>0.05	25.7 ± 4.1	25.6 ± 4.1	P>0.05	15 (38%)	12 (40%)	N/A
[3]	59.4 ± 11.4	59.7 ± 11.8	N/A	20 (90.9%)	18 (90.0%)	N/A	N/A	N/A	N/A	6 (27.3%)	0	N/A
[4] A	56.53 ± 9.67	55.22 ± 9.43	0.642	19 (55.9%)	9 (50.0%)	0.686	N/A	N/A	N/A	9 (26.5%)	0	0.016
B	57.94 ± 9.70	57.69 ± 10.86	0.844	112 (73.7%)	51 (48.6%)	p<0.001	N/A	N/A	N/A	26 (17.1%)	9 (8.6%)	0.050
[5]	65.90±4.00	59.83±3.15	p>0,05	14 (48.3%)	16 (53%)	N/A	29.97±1.90	25.30±1.47	p<0,05	N/A	N/A	N/A
[6]	61±9.74	60±9.77	p=0.094	161 (75,2%)	75 (40,3%)	p< 0.00001	24.54±35	24.41±6.59	N/A	N/A	N/A	N/A
[7]	64.1±8.6	61.6±10.0	0.17	32 (60.4%)	30 (56.6%)	0.69	29.4±5.9	29.1±5.7	0.76	15 (28.3%)	3 (5.7%)	0.002
[8]	68.27 ± 9.54	66.14 ± 11.41	N/A	15 (51.72)	18 (51.43)	N/A	23.54 ± 3.69	23.70 ± 2.60	N/A	N/A	N/A	N/A
[9]	61.1±9.4	58.7±7.3	P>0.05	33 (85%)	39 (78%)	P>0.05	25.7±4.1	22.4±2.4	P<0.01	15 (38%)	1 (2%)	P<0.01
[10]	N/A	N/A	N/A	N/A	N/A	N/A	N/A	N/A	N/A	N/A	N/A	N/A
[11]	58.84 ± 1.52	36.64 ± 3.53	<0.001	36 (78,3%)	10 (58,8%)	0.384	24.59 ± 0.75	20.45 ± 0.91	0.017	N/A	N/A	N/A
[12]	63.6±7.2	62.9±6.8	P>0.05	27 (90%)	23 (77%)	P>0.05	25.1±2.8	24.8±4.1	P>0.05	11 (37%)	12 (40%)	P>0.05
[13]	73.1±10.4	63.4±3.4	P<0.05	10 (91%)	7 (70%)	P>0.05	24.0 ±3.1	24.3±4.2	P>0.05	5 (45%)	2 (20%)	P>0.05
[14]	63.63	60.01	N/A	30 (43%)	41 (42%)	N/A	24.34	23.83	N/A	N/A	N/A	N/A
[15]	53.3 ± 6.7	41.5 ± 9.6	P<0.05	18 (100%)	9 (75%)	P<0.05	26.5 ± 5.4	25.2 ± 3.7	P>0.05	N/A	N/A	N/A
[16]	58.3	57.06±9.23	P>0.05	117 (83%)	19 (39%)	P<0.001	N/A	N/A	N/A	50 (35,5%)	13 (27.08)	P>0.05
[17]	54.92 ± 8.51	52.65 ± 8.79	P>0.05	48 (80%)	22 (73.3%)	P>0.05	25.48 ± 2.42	24.39 ± 2.86	P>0.05	N/A	N/A	N/A
[18]	N/A	N/A	N/A	N/A	N/A	N/A	N/A	N/A	N/A	N/A	N/A	N/A
[19]	65.4±9.3	48.2±16.3	P<0.0001	67 (76%)	46 (40%)	P<0.0001	31.2±5.9	27.4±5.8	P<0.0001	23 (26%)	2 (2%)	P<0.0001
[20]	64.1± 7.7	62.4± 10.5	0.08	124 (73%)	108 (65%)	0.1	30.52± 5.3	28.46± 4.73	0.001	50 (30%)	16 (15%)	0.003
[21]	65.15 ± 11.53	73.20 ± 9.06	< 0.001	N/A	N/A	N/A	26.43	24.47	N/A	N/A	N/A	N/A
[22]	67.2 ±9	57 ± 11.1	p > 0.5	11 (73,3%)	5 (33,3%)	N/A	29.4± 5.05	25.95± 4.21	p < 0.05	N/A	N/A	N/A
[23]	61.7 ± 9.0	61.4 ± 7.5	P>0.05	38 (84,4%)	13 (68.4%)	P>0.05	24.7 ± 2.4	24.2 ± 4.3	P>0.05	7 (15.6%)	9 (47.4%)	P>0.05

1. Alhmoud, T., Kumar, A., Lo, C.C., Al-Sadi, R., Clegg, S., Alomari, I., Zmeili, T., Gleasne, C.D., McMurry, K., Ko Dichosa, A.E., Vuyisich, M., Chain, P.S., Mishra, S., Ma, T. (2019). Investigating intestinal permeability and gut microbiota roles in acute coronary syndrome patients. *Human Microbiome J., 13*:100059. https://doi.org/10.1016/j.humic.2019.100059.
2. Emoto, T., Yamashita, T., Kobayashi, T., Sasaki, N., Hirota, Y., Hayashi, T., So, A., Kasahara, K., Yodoi, K,. Matsumoto, T., *et al*. (2017). Characterization of gut microbiota profiles in coronary artery disease patients using data mining analysis of terminal restriction fragment length polymorphism: gut microbiota could be a diagnostic marker of coronary artery disease. *Heart Vessels*, *32*(1):39–46. https://doi.org/10.1007/s00380-016-0841-y.
3. Kwun, J.S., Kang, S.H., Lee, H.J., Park, H.K., Lee, W.J., Yoon, C.H., Suh, J.W., Cho, Y.S., Youn, T.J., Chae, I.H. (2022). Comparison of thrombus, gut, and oral microbiomes in Korean patients with ST-elevation myocardial infarction: a case–control study. *Exp. Mol. Med*., *52*(12):2069–2079. https://doi.org/10.1038/s12276-020-00543-1.
4. Zheng, Y.Y., Wu, T.T., Liu, Z.Q., Li, A., Guo, Q.Q., Ma, Y.Y., Zhang, Z.L., Xun, Y.L., Zhang, J.C., Wang, W.R., *et al*. (2020). Gut microbiome-based diagnostic model to predict coronary artery disease. *J. Agric. Food Chem*., *68*(11):3548–3557. https://doi.org/10.1021/acs.jafc.0c00225.

   A- Zhengzhou cohort, B- Xinjiang cohort.
5. Ivashkin, V.T., Kashukh, Y.A. (2019). Impact of L-carnitine and phosphatidylcholine containing products on the proatherogenic metabolite TMAO production and gut microbiome changes in patients with coronary artery disease. *Voprosy Pitaniia*, *88*(4):25–33. https://doi:10.24411/0042-8833-2019-10038.

6. Jacobson, G., McKinnon, M., Miller, I., Stayte, L., Thaneeru, P., Bird, S., Devlin, G. (2020). Association of gut and oral microbiomes in acute coronary syndrome with blood markers of gut leakage, inflammation and coronary artery disease risk. *Heart. Lung. Circ.*, *29*(Suppl.1):S14. https://doi.org/10.1016/j.hlc.2020.05.037.
7. Toya, T., Corban, M.T., Marrietta, E., Horwath, I.E., Lerman, L.O., Murray, J.A., Lerma, A. (2020). Coronary artery disease is associated with an altered gut microbiome composition. PLoS One, 15(1):e0227147. https://doi:10.1371/journal.pone.0227147.
8. Cui, L., Zhao, T., Hu, H., Zhang, W., Hua, X. (2017). Association study of gut flora in coronary heart disease through high-throughput sequencing. *BioMed. Res. Int.*, *2017*:1–10. https://doi.org/10.1155/2017/3796359.
9. Emoto, T., Yamashita, T., Sasaki, N., Hirota, Y., Hayashi, T., So, A., Kasahara, K., Yodoi, K., Matsumoto, T., Mizoguchi, T., *et al*. (2016). Analysis of gut microbiota in coronary artery disease patients: a possible link between gut microbiota and coronary artery disease. *J Atheroscler. Thromb*., *23*(8):908–921. https://doi.org/10.5551/JAT.32672.
10. Jie, Z., Xia, H., Zhong, S.L., Feng, Q., Li, S., Liang, S., Zhong, H., Liu, Z., Gao, Y., Zhao, H., *et al*. (2017). The gut microbiome in atherosclerotic cardiovascular disease. *Nat. Commun., 8*(1):845. https://doi:10.1038/s41467-017-00900-1.
11. Liu, Z., Li, J., Liu, H., Tang, Y., Zhan, Q., Lai, W., Ao, L., Meng, X., Ren, H., Xu, D., Zeng, Q. (2019). The intestinal microbiota associated with cardiac valve calcification differs from that of coronary artery disease. *Atherosclerosis*, *284*:121–128. https://doi.org/10.1016/j.atherosclerosis.2018.11.038.
12. Yoshida, N., Sasaki, K., Sasaki, D., Yamashita, T., Fukuda, H., Hayashi, T., Tabata, T., Osawa, R., Hirata, K., Kondo, A. (2019). Effect of resistant starch on the gut microbiota and its metabolites

inpatients with coronary artery disease. *J. Atheroscler. Thromb*., *26*(8):705–719. https://doi.org/10.5551/jat.47415.

13. Yoshida, N., Emoto, T., Yamashita, T., Watanabe, H., Hayashi, T., Tabata, T., Hoshi, N., Hatano, N., Ozawa, G., Sasaki, N., et al. (2018). Bacteroides vulgatus and Bacteroides dorei reduce gut microbial lipopolysaccharide production and inhibit atherosclerosis. Circulation, 138(22):2486–2498. https://doi:10.1161/CIRCULATIONAHA.118.033714.
14. Zhu, Q., Gao, R.,, Zhang Y., Pan, D., Zhu, Y., Zjang, X., Yang, R., Jiang, R., Xu, Y., Qi, H. (2018). Dysbiosis signatures of gut microbiota in coronary artery disease. *Physiol Genomics., 50*(10):893–903. https://doi:10.1152/physiolgenomics.00070.2018.
15. Liu, F., Fan, C., Zhang, L., Li, Y., Hou, H., Ma, Y., Fan, J., Tan, Y., Wu, T., Jia, S., Zhang, Y. (2020). Alterations of gut microbiome in Tibetan patients with coronary heart disease. *Front. Cell Infect. Microbiol*., *10*:373. https://doi.org/10.3389/FCIMB.2020.00373.
16. Dong C, He Z, Zhu Q, Liu J, Gao F, Li K, Sun S, Liu Q, Wang Y, Tang Y, Hu S, Zhu H. Correlation Network Analyses Based on Metagenomics and Multi-type Metabolomic Data Identified Biomarkers of Coronary Artery Disease. doi:10.21203/rs.3.rs-65514/v1. PPR:PPR210556.
17. Gao, J., Yan, K.T., Wang, J.X., Dou, J., Wang, J., Ren, M., Ma, J., Zhang, X., Liu, Y. (2020). Gut microbial taxa as potential predictive biomarkers for acute coronary syndrome and post-STEMI cardiovascular events. Sci. Rep., 10(1):2639. https://doi.org/10.1038/s41598-020-59235-5.
18. Pisano, E., Severino, A., Bugli, F., Pedicino, D., Sterbini, F.P., Martini, C., Vinci, R., Canonico, F., Bonanni, A., D'Aiello, A., et al. (2020). Plaque instability in acute coronary syndromes: a possible pathogenic role of gut microbial communities. Eur. Heart J., 41(Suppl.2):2020,ehaa946.1254. https://doi.org/10.1093/ehjci/ehaa946.1254.

19. Toya, T., Ozcan, I., Corban, M.T., Sara, J.D., Marietta, E.V., Ahmad, A., Horwath, I.E., Loeffler, D.L., Murray, J.A., Lerman, L.O., Lerman A. (2021). Compositional change of gut microbiome and osteocalcin expressing endothelial progenitor cells in patients with coronary artery disease. *PLoS One*, *16*(3):e0249187. https://doi.org/10.1371/journal.pone.0249187.

20. Sawicka-Śmiarowska, E., Bondarczuk. K., Bauer, W., Niemira, M., Szalkowska, A., Raczkowska, J., Kwasniewski, M., Tarasiuk, E., Dubatowska, M., Lapinska, M., *et al.* (2021). Gut microbiome in chronic coronary syndrome patients. *J. Clin. Med.*, *10*(21):1–20. https://doi.org/10.3390/jcm10215074.

21. Chiu, F.C., Tsai, C.F., Huang, P.S., Shih, C.Y., Tsai, M.H., Hwang, .JJ., Wang, Y.C., Chuang, E.Y., Tsai, C.T., Chang, S.N. (2022). The gut microbiota and seleno-compound as emerging risk factors for acute myocardial infarction. *J. Clin. Med.*, *11*(5):1462. https://doi.org/10.3390/jcm11051462.

22. Choroszy, M., Sobieszczanska, B., Litwinowicz, K., Łaczmanski, Ł., Chmielarz, M., Roleder, T., Radziejewska, J., Wawrzynska, M. (2022). Co-toxicity of bacterial metabolites, to vascular endothelial cells in coronary arterial disease accompanied by gut dysbiosis. *Nutrients, 14*:424. https://doi:10.3390/nu14030424.

23. Hu, J.L., Yao, Z.F., Tang, M.N., Tang, C., Zhai, X.F., Su, X., Lu, D.B., Li, Q.R., Wang, Z.S., Yan, Y., Wang, Z. (2021). Gut microbiota community shift with severity of coronary artery disease. *Engineering*, *7*(12):1715–1724. https://doi.org/10.1016/j.eng.2020.05.025.
